# Role of Environmental Toxicants on Neurodegenerative Disorders

**DOI:** 10.3389/ftox.2022.837579

**Published:** 2022-05-11

**Authors:** Masarat Nabi, Nahida Tabassum

**Affiliations:** ^1^ Department of Environmental Science, University of Kashmir, Srinagar, India; ^2^ Department of Pharmaceutical Sciences, University of Kashmir, Srinagar, India

**Keywords:** alzheimer’s disease, environmental toxicant, neurodegenration, neurotoxins, Parkinson’s disease

## Abstract

Neurodegeneration leads to the loss of structural and functioning components of neurons over time. Various studies have related neurodegeneration to a number of degenerative disorders. Neurological repercussions of neurodegeneration can have severe impacts on the physical and mental health of patients. In the recent past, various neurodegenerative ailments such as Alzheimer’s and Parkinson’s illnesses have received global consideration owing to their global occurrence. Environmental attributes have been regarded as the main contributors to neural dysfunction-related disorders. The majority of neurological diseases are mainly related to prenatal and postnatal exposure to industrially produced environmental toxins. Some neurotoxic metals, like lead (Pb), aluminium (Al), Mercury (Hg), manganese (Mn), cadmium (Cd), and arsenic (As), and also pesticides and metal-based nanoparticles, have been implicated in Parkinson’s and Alzheimer’s disease. The contaminants are known for their ability to produce senile or amyloid plaques and neurofibrillary tangles (NFTs), which are the key features of these neurological dysfunctions. Besides, solvent exposure is also a significant contributor to neurological diseases. This study recapitulates the role of environmental neurotoxins on neurodegeneration with special emphasis on major neurodegenerative disorders such as Alzheimer’s and Parkinson’s disease.

## 1 Introduction

Neurodegenerative disorders usually affect both mental and physical abilities (Bonaz et al., 2021). The majority of neurodegenerative disorders are inextricably related to ageing with the likelihood of growing progressively as one gets older (Leak, 2014). The average life expectancy has increased dramatically as a result of medical advancements (Shang and Goldman, 2008). Therefore, the incidence of neurological disorders is projected to become more prevalent as the population ages (Wittchen et al., 2011). The majority of such disorders still have two fundamental concerns: 1) identifying the etiology in the majority of cases and 2) developing therapeutic drugs (Stocchi and Olanow, 2013). Unfortunately, despite much research, none of these difficulties have been addressed. Environmental factors have long been thought of as contributing to neurodegenerative disorders (Nakamura and Lipton, 2009). This is evident in the case of Parkinson’s disease, where epidemiological research has attributed environmental factors to the condition (Tysnes and Storstein, 2017). Moreover, several neurotoxicants found in the environment cause behavioural and clinical symptoms in people with Parkinson’s disease (Uversky, 2004), although none of them have been linked to the development of such neurological conditions. Owing to a large number of chemicals and the diversity in human exposure throughout a lifetime, establishing such connections will be challenging. Finally, the primary objective of investigating the impact of neurotoxin exposures in neurodegenerative disorders is to detect significant risk factors (Migliore and Coppedè, 2009). The prevalence and severity of neurodegenerative disorders on human wellbeing may be reduced by removing recently identified risk factors (World Health Organization, 2006). The relationship between neurotoxins and neurodegenerative disorders allows for the development of novel *in-vivo* models and a better knowledge of the pathological processes of human ailments (Bezard et al., 2013). Our understanding of pathogenic processes, on the other hand, has significantly improved, and this information will be critical in pinpointing both the etiology as well as devising novel therapeutics (Copf, 2016; Cacace et al., 2016). Improved and more realistic *in-vivo* models are critical for the screening and formulation of innovative treatments that will possibly be produced to manage this dreadful set of illnesses.

### 1.1 Alzheimer’s Disease

Alzheimer’s disorder is primarily regarded as a condition of degenerative ageing, with the majority of instances diagnosed in individuals aged 65 and over (Park et al., 2005). Alzheimer’s disease is so far the most prevalent neurological disorder (Bauer et al., 2021). It is a severe condition with dementia as a characteristic symptom (Guest et al., 2020). Multiple theories have been suggested to address the etiology of Alzheimer’s disease, such as genetic abnormalities, oxidative stress (Decourt et al., 2021), β-amyloid toxicity, and environmental factors (Salahuddin et al., 2021). Alzheimer’s disease or an associated type of dementia affects roughly 50 million individuals worldwide, with that figure estimated to rise to 152 million by 2050 (Thapa et al., 2020). Alzheimer’s disease affects more than 5.8 million individuals in the United States, with 13.8 million Americans over 65 estimated to be affected by the disease by 2050 (Calderón-Garcidueñas et al., 2019). Environmental factors have a role in the development of Alzheimer’s disease, which is characterized by gradual impairments in cognitive, learning capacity, memory function, and executive thinking (Więckowska-Gacek et al., 2021). The average lifespan for patients with Alzheimer’s disease ranges from 3–10 years. Since age is the most important determinant of life span, therefore patients diagnosed with diseases in their 60s and early 70s have an average life expectancy of 7–10 years, whereas patients diagnosed with difficulties in their 90s have an average life span of 3 years or less (Zanetti et al., 2009). Early-onset instances also exist and are often caused by hereditary factors (van der Flier et al., 2011; Zou et al., 2014). A plethora of studies have been conducted in recent years to investigate the role of environmental elements including solvents, heavy metals, and pesticides in the neurodegenerative process.

### 1.2 Parkinson’s Disease

Parkinson’s disease is a prevalent neurological illness, next to Alzheimer’s disease in terms of global prevalence (Song et al., 2020). Parkinson’s disease is more common as individuals become older, although around 4% of individuals with the condition are identified before they are 50. Men are 1.5 times more likely than women to get Parkinson’s disease (Edinoff, et al., 2020). It affects over 10^7^ individuals globally. Parkinson’s disease affects almost one million individuals in the United States, which is higher than the total number of persons identified with disorders like multiple sclerosis, down’s syndrome, and amyotrophic lateral sclerosis (Fritsch et al., 2012). By 2030, this number is predicted to reach 1.2 million (Tanner, 2020). Annually, over 60,000 Americans are identified with Parkinson’s disease (Chakraborty et al., 2020). Motor symptoms such as bradykinesia with stiffness (hypomimia), resting tremor, freezing, festination, dyskinesia, dystonia, sialorrhea, micrographia shuffling gait, and hypophonia along with postural instability at a later phase, define Parkinson’s disease (Rawat and Pandey, 2022). Non-motor symptoms such as dementia, depression, seborrheic dermatitis, excessive sweating, orthostatic hypotension, weight loss, hallucinations, delusions, and autonomic dysfunction may also be present (Goldman and Guerra, 2020). Thus, neurodegenerative disorders such as Alzheimer’s disease and Parkinson’s disease are becoming more common as the global population ages (Cummings, 2017). Among the major contributors are air pollutants, heavy metals for instance lead, arsenic, manganese, aluminium, etc, rapid urbanization, industrialization, and extensive use of synthetic chemicals such as solvents, pesticides, fungicides, and herbicides (Vardhan et al., 2019). This review provides an analysis of the role of various environmental toxicants on neurodegenerative disorders such as Alzheimer’s and Parkinson’s disease.

## 2 Impact of Environmental Toxicants on Neurodegenerative Disorders

**FIGURE 1:**
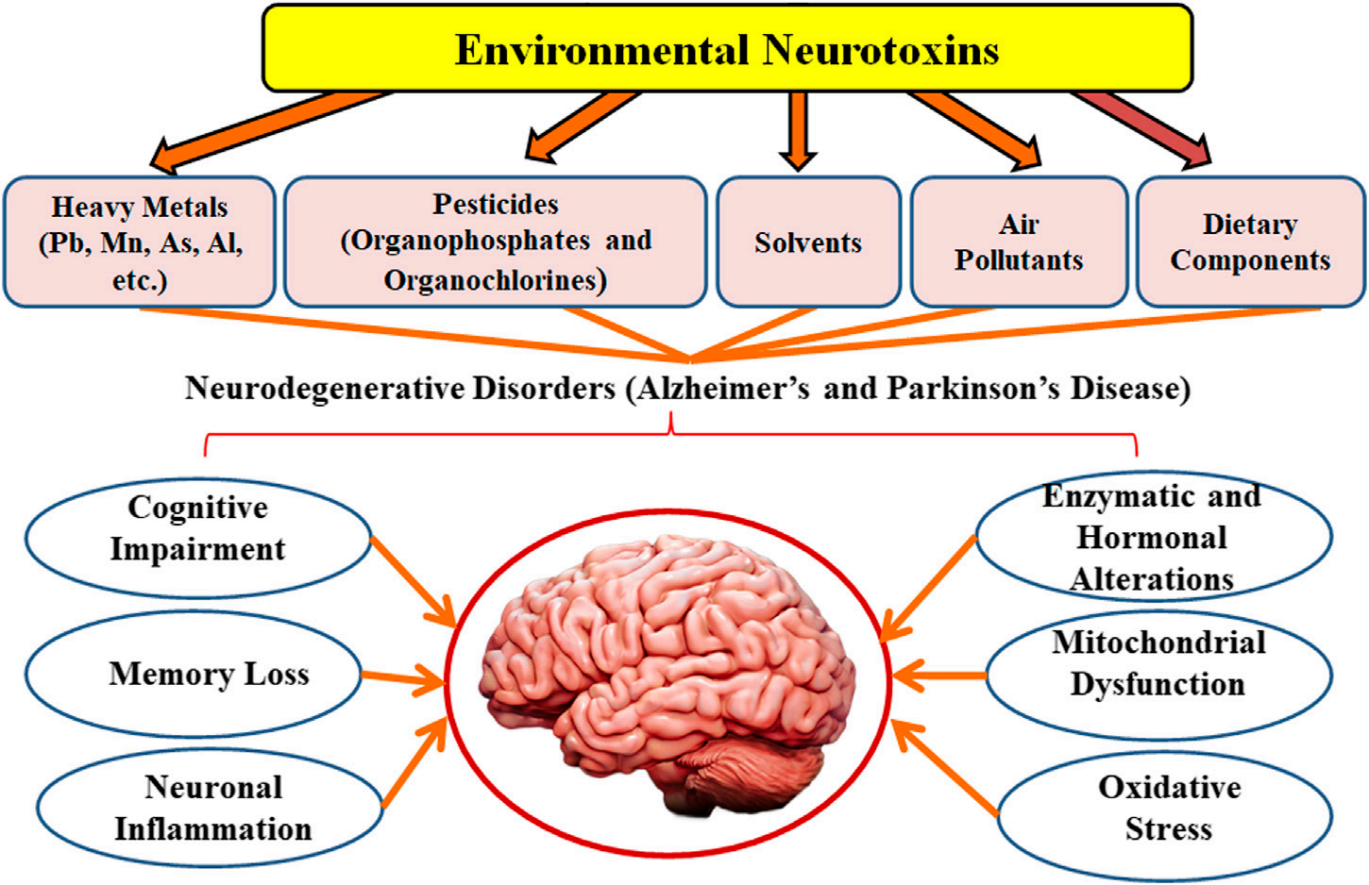
The impact of environmental toxicants on neurodegenerative disorders.

Environmental pollution is a severe problem that has been linked to a higher prevalence of sickness and fatalities around the globe (Zar and Ferkol, 2014). Air contaminants are the primary cause of lung and brain inflammation, which disrupts the proper functioning of the central nervous system (CNS) (Block and Calderón-Garcidueñas, 2009). Atmospheric pollutants cause CNS pathology by causing oxidative stress, microglial cells activation, neuronal inflammation, and changes in blood-brain membrane permeability (Calderón-Garcidueñas et al., 2008). Likewise, heavy metal toxicity has severe and long-term consequences on the brain, resulting in cognitive impairment (Ortega et al., 2020). Chronic exposure to heavy metals may interrupt the development of physical, muscular, and neurological conditions that resemble diseases such as Parkinson’s and Alzheimer’s disorders (Nabi, 2021). Pesticides, on the other hand, have a significant impact on the etiology of neurodegenerative illnesses (Parrón et al., 2011; Chin-Chan et al., 2015; Mostafalou and Abdollahi, 2018). Traditional therapies are efficacious but often do not have optimal clinical efficiency towards such problems, developing a treatment approach for environmental pollutants-induced neurodegenerative diseases is a difficult challenge. The impact of environmental toxicants on neurodegenerative disorders is presented in Table 1 and Figure 1.

**TABLE 1:** Effect of various environmental toxins on neurodegenerative disorders.

S. No.	Environmental Toxin	Model Used	Duration/Dose	Effect	References
1	Trichloroethylene (TCE)	Elderly rats	200 mg/kg	Nigrostriatal dopaminergic impairment, elevated oxidative stress, induced endolysosomal impairment, and α-synuclein deposition	De Miranda et al. (2021)
2	Organophosphate pesticide [chlorpyrifos (CPF)]	Rat (males and females)	20 months	CPF exposure caused chronic microglial dysregulation and accelerated neurodegeneration in both males and females	Voorhees et al. (2019)
3	Aluminium chloride (AlCl_3_)/aluminium lactate (Al (lac)_3_)	Mice	3 months	Acetylcholinesterase activation	Zatta et al. (2002)
4	Inorganic arsenic (iAs)	3xTgAD mouse	6 months	Decreased ATP content via the decline of complex-I levels, and increased ROS production in the hippocampus, greater immune-positive responses to amyloid isoforms and phosphorylated tau were seen in the frontal cortex and hippocampus	Niño et al. (2018)
5	Lead acetate (PbA)	Pregnant Wistar female rats	2 weeks/15 mg/kg	Production of pro-inflammatory cytokines such as IL-1 and TNF-α in the hippocampus and IL-6 in the forebrain of immature rat brain	Balali-Mood et al. (2021)
6	Chlorpyrifos (CPF)	Adult male rats (Long Evans)	21 days/3 and 10 mg/kg/day	Cortical Acetylcholinesterase (AChE) suppression, hippocampus AChE suppression, whole blood ChE reduction, transcriptome alterations in genes producing hippocampal neuropeptides such as brain-derived neurotrophic factor (BDNF), cortistatin (CORT), and neuropeptide Y (NPY)	Lee et al. (2016)
7	Methoxychlor (MXC)	Adult female CD1 mice	20 days/16, 32, or 64 mg/kg/day	Decreased striatal dopamine, dopamine transporter vesicular monoamine transporter-2 levels, and elevated protein carbonyl levels in non-synaptic mitochondria	Schuh et al. (2009)
8	Dichlorvos (organophosphate)	Rat	12 weeks/6 mg/kg/day	Increased mitochondrial Ca^2+^ absorption, reduced cytochrome oxidase (complex-IV) electron transfer activities, and modified mitochondrial complex-I, and complex- II activity increase in malondialdehyde, protein carbonyl, and 8-hydoxydeoxyguanosine synthesis, mtDNA oxidation, and oligonucleosomal DNA fragmentation	Kaur et al. (2007)
9	PCBs	Rat	14 days	Altered DA neurochemistry, DAergic protein downregulation, elevated oxidative stress, neuronal injury and the deterioration of both VM and striatal GABA neurons, prior to the death of VM DA neurons	Lyng et al. (2007)
10	PCBs blend	Female Sprague-Dawley rats	-	Impaired memory, anxiety-like behaviour, substantially lower white blood cell counts, slightly affected plasma metabolomics, and impacted transcription brain activity, with 274 genes upregulated and 58 genes downregulated	Wang et al. (2022)
11	Simulated vehicle exhaust exposure (SVEE)	Adult male Sprague Dawley rats	2 weeks (5 h/day)	Behavioural and cognitive abnormalities, elevated oxidative stress, decreased antioxidant response, and mitochondrial impairment	(Salvi et al. (2020)
12	Indoor nanoscale particulate matter (INPM)	3D human organotypic model	-	INPM aggravated inflammation caused by ROS and stimulated abnormal expression of the nuclear transcription factor Nrf2 following ROS accumulation. Disruption of γ-glutamate synthase (γ-GCS) and heme oxygenase (HO-1) synthesis, exacerbating the antioxidant system’s imbalance and thereby influencing BBB bio-function by Keap1-Nrf2-ARE pathways	Li et al. (2020)
13	Triphenyl phosphate (TPP) and diphenyl phosphate (DPP)	Weaned male mice (C57/BL6)	30 days/(0, 50, or 150 mg/kg/day)	Thalamus and hippocampus inflammation. Changes in glutamic acid, N-acetyl CoA metabolites, and organic acid levels. Interference with amino acid, lipid metabolism, brain transcription and cell death processes (FOXO and MAPK signalling pathways). Upregulation of Anti-inflammatory cytokines such as TNF-α and interleukin-6 (IL-6) and downregulation of antioxidant genes such as nuclear factor-E2-related genes	Liu et al. (2020)
14	Rotenone	SH-SY5Y cell lines	-	Increased production and structural alterations in oligomers and fibrils	Srivastava et al. (2020)
15	Paraquat	Mice	-	Promoted oxidative stress, degenerative cellular apoptosis in the SNpc, striatum, and cerebellum, as well as dopamine deficiency in the SNpc and striatum, which led to motor and cognitive impairments	Onyeka et al. (2022)
16	PM_2.5_	SPF male C57BL/6 J mice	Seven days	Cognitive deficits, loss of neurons, protein aggregates were detected	Liu et al. (2019)
17	DDT and DDE	Human neuroblastoma cells	-	Increased amyloid precursor protein levels	Richardson et al. (2014)
18	Al and Hg	Human neuronal-glial (HNG) cells	-	Large upsurge in pro-inflammatory signalling mechanisms via notable induction of NF-kB (p50/p65) in response to Al and Hg individually or in combination	Alexandrov et al. (2018)
19	Pb and Mn	Male and female Sprague Dawley rats	Pb (10 mg/ml), Mn (2 mg/ml) or a mixture	Low levels of Pb and Mn produced gender-specific neurological impairments	Betharia and Maher, (2012)
20	Aluminium chloride (AlCl_3_)/aluminium lactate (Al (lac)_3_)	Acetylcholinesterase Assay (mouse brain homogenates)	-	Acetylcholinesterase activation	Zatta et al. (2002)
21	Arsenite	Cultures of primary astrocytes	24 h	Increased glutamate-induced astrocytic calcium levels, increased levels of d-serine, γ-aminobutyric acid and glycine, affected glutamate-induced gliotransmitter release from astrocytes and disturbed neuronal function	Wang et al. (2012)
22	Sodium arsenite	lymphocytes	72 h	Impaired GLUT1 trafficking and function via calpain dysregulation	Pánico et al. (2019)
23	Arsenate	Primary astrocyte culturesfrom the brains of newborn Wistar rats	-	Rapid GSH export stimulation, MRP1 inhibition prevented arsenate induced GSH export	Meyer et al. (2013); Dringen et al. (2015)
24	Arsenite	Primary astrocyte cultures from rat brain	-	Rapid GSH export stimulation, MRP1 inhibition prevented arsenite-induced GSH export, glycolytic lactate production stimulation	Tadepalle et al. (2014); Dringen et al. (2015)
25	Trichloroethylene (TCE)	Aged rats	200 mg/kg	Elevated oxidative stress, caused endolysosomal dysfunction, protein accumulation (α-synuclein) and induced LRRK2 kinase activity which resulted in the selective dopaminergic neurotoxicity	Castro et al. (2020)
26	Manganese	Children	-	Inferior intelligence quotient (IQ) scores	Roels et al. (2012)
27	Tau phosphorylation, and metal ions (Al^3+^ and Fe^3+^)	confocal single-particle fluorescence method	-	Tau phosphorylation and Al^3+^ and Fe^3+^ increased both the production of blended oligomers and the incorporation of α-synuclein into pre-formed tau oligomers	Nübling et al. (2012)
28	Metal composites (arsenic, manganese, and lead)	Rat	-	Reduced rat motor indices	Sanders et al. (2015)
29	Dietary habits	Human males (49,692) and females (81,676)	16 years of follow-up	Two eating trends emerged such as prudent and western. The prudent dietary pattern was found to be inversely related to the prevalence of Parkinson’s disease, but not the western pattern	Gao et al. (2007)

### 2.1 Air Contaminants

Air pollution has been linked to neurodegenerative disorders such as Alzheimer’s disease, Parkinson’s disease, and other neurological conditions (Genc et al., 2012; Bandyopadhyay, 2016; Costa et al., 2020). Several air pollutants, including nanoparticles, have been found to rapidly translocate to the CNS and may stimulate innate immune responses (Block and Calderón-Garcidueñas, 2009). Recent findings demonstrate that atmospheric contaminant-induced neuroinflammation, oxidative stress, microglial activation, cerebrovascular impairment, and changes in the blood-brain barrier all contribute to CNS disorders (Lee et al., 2017). Particulate matter, gases such as ground-level ozone, carbon monoxide, sulphur oxides, and nitrogen oxides are all examples of air pollutants that may be present in both indoor and outdoor atmospheres (Genc et al., 2012). The most prevalent and hazardous components appear to be particulate matter and ground-level ozone, which are primarily produced by nitrogen oxides and volatile organic compounds. Particulate matter is a mixture of solid particles and liquid droplets suspended in the air that is particularly detrimental to the CNS (Liu et al., 2015). The Particulate matter having a diameter of 2.5 µm (PM_2.5_) is mainly comprised of both organic and inorganic substances, such as sulphates, nitrates, carbon, ammonium, hydrogen ions, lipopolysaccharide, metals, and water (Wang et al., 2018). Oil refineries, metal processing industries, tailpipe and brake emissions, domestic fuel burning, power plants, and wildfires are all primary sources of PM_2.5_ particles (Xie et al., 2021). The blood-brain barrier and olfactory neurons are two key mechanisms for PM_2.5_ to penetrate the CNS (Shou et al., 2019). PM_2.5_ toxicity in the brain is caused by two main pathways: 1) the inflammatory response and 2) oxidative stress (Zhang et al., 2018; Wang et al., 2021). The neuroinflammatory process is triggered when PM_2.5_ activates microglia inappropriately. PM_2.5_ has been related to neurodegenerative diseases such as Alzheimer’s and Parkinson’s disease in several epidemiological (Zanobetti et al., 2014; Kioumourtzoglou et al., 2016; Rhew et al., 2021) and toxicological studies (Wang et al., 2021). However, limited studies have been conducted on the *in-vivo* and molecular pathways of neurotoxicity caused by PM_2.5_ exposure. In this context, Liu et al. (2019) explored the link between PM_2.5_ exposure and neurodegeneration. For 7 days, specific-pathogen-free (SPF) male C57BL/6 J mice were intranasally exposed to PM_2.5_ at a dosage of 0.193 mg/kg/day. Acute PM_2.5_ exposure led to various changes, including cognitive impairments, neuronal death, protein aggregation, and so on. Oxidative damage and inflammation may have an impact on these damaging pathways.

### 2.2 Dietary Factors

Diet is one of the environmental components that influence Alzheimer’s (Hu et al., 2013) and Parkinson’s disorders (Agim and Cannon, 2015), for instance, a hyperlipidemic diet may promote β-amyloid deposition (Hu et al., 2020). Calorie consumption, caffeine, alcohol, and metals absorbed through food and lipids, among other factors, have been linked to Alzheimer’s related parameters such as epigenetic, β-amyloid, tau proteins, oxidative stress, and oxygen reactive species (ROS) (Fernández-Sanz et al., 2019). Several *in-vitro*, *in-vivo*, and human epidemiological investigations have demonstrated a range of dietary components that influence the incidence of Parkinson’s and Alzheimer’s disease (Rossi et al., 2008; Vauzour et al., 2010). Gao et al. (2007) investigated relationships between dietary habits and the risk of Parkinson’s disease in the health professional’s follow-up study (1986–2002) and the nurse’s health study (1984–2000). The principal components analysis was applied to identify main diet trends and the Alternate Healthy Eating Index (AHEI) and the alternate Mediterranean diet score (aMed) to measure dietary quality in 49,692 males and 81,676 females who were free of Parkinson’s disease at the beginning of the study. Within each cohort, relative risks were calculated using Cox proportional hazards models and then aggregated using a random-effects model. After 16 years of follow-up, they discovered 508 Parkinson’s disease cases. Two eating trends emerged from the analysis of main components: prudent and western. The prudent dietary pattern, marked by high consumption of fruit, vegetables, and fish, was found to be inversely related to the prevalence of Parkinson’s disease, but not the Western pattern. As a result, researchers concluded that a diet rich in fruits, vegetables, legumes, whole grains, nuts, fish, and poultry, as well as a reduced saturated fat consumption and limited alcohol use, may help prevent Parkinson’s disorder (Gao et al., 2007).

### 2.3 Heavy Metals

Intrinsic bio-metals like copper, iron, and zinc, as well as extrinsic metals like aluminium, potentially play a significant role as factors/cofactors in the etiology of various neurological disorders for instance Alzheimer’s disease (Zatta et al., 2002). Whereas, heavy metals including lead, cadmium, arsenic, and manganese are often used and possibly lead to neurological conditions namely*,* Parkinson’s and Alzheimer’s disease by elevating neuronal oxidative stress, mitochondrial dysfunction, inflammatory processes, and apoptosis (Chen et al., 2016; Karri et al., 2016). Some of the metals associated with neurodegeneration are discussed below.

#### 2.3.1 Aluminium

Aluminium is among the most prevalent and widespread metals in our environment (Hardisson et al., 2017). It is widely used in commercial as well as domestic applications (kitchenware) Gándara, (2013). Humans are exposed to aluminium from various sources, such as diet, which accounts for 95% of body aluminium, drinking water, air, cosmetics, and medical drugs, primarily antacids (Skalny et al., 2021). Its uptake primarily takes place *via* food, breathing aluminium dust, and skin contact (Jaishankar et al., 2014). Various researches have revealed the possibility for aluminium neurotoxicity, however, the exact mechanism of its toxicity is unclear. It is a well-known neurotoxin that has been linked to the development of neurological disorders such as dementia (Kumar and Gill, 2009), encephalopathy, seizures, motor neuron degeneration, Alzheimer’s disease (Reusche, 2003; Tomljenovic, 2011), Parkinson’s disease, and so on (Polizzi et al., 2002). Its neurotoxicity has been linked to increased oxidative stress, amyloid precursor protein (APP) production, β-amyloid accumulation, and decreased cholinergic projections (Maya et al., 2016). It causes neurotoxicity in primary brain cells via increasing iron deposition and the formation of ROS, which impacts a wide range of signalling pathways and eventually leads to apoptotic cell death (Verstraeten et al., 2008). Furthermore, the molecular and epidemiological research in Alzheimer’s disease point to an aberrantly localized deposition of aluminium in the brain (Miu and Benga, 2006). It may disrupt several metabolic functions, notably acetylcholine metabolism and the breakdown of amyloid peptides, and so may serve as an etiopathogenic co-factor. In addition, various investigations have demonstrated the effect of aluminium on acetylcholine metabolism (Jankowska et al., 2000), and the distinctive neurotoxicity of aluminium has been documented in a range of biological models (Klotz et al., 2017). The influence of metal ions on acetylcholinesterase (AChE) activity has been examined in both *in-vivo* as well as *in-vitro* models (Gill et al., 1991; Senger et al., 2011; de Lima et al., 2013). In this connection, Zatta et al. (2002) investigated both *in-vitro* and *in-vivo* models to explore the effects of aluminium on the activity of mouse brain AChE. Aluminium chloride (AlCl_3_) or aluminium lactate (Al (lac)_3_) was orally administered in mice, and the findings demonstrated that *in-vivo* treatment increased the AChE activity. An activating effect was revealed *in-vitro* when aluminium compounds were directly administered into mouse brain homogenates. However, the activation detected *in-vivo* was significantly higher than that recorded *in-vitro*. Furthermore, the activation-induced via Al (lac)_3_ was greater than that attained after treatment with aluminium chloride.

#### 2.3.2 Arsenic

Inorganic arsenic (iAs) is a strong environmental toxin found in soil, air, and water (Hughes, 2006), where it is a widespread contaminant from both human and natural causes (Ng, 2005). Arsenic-tainted drinking water affects roughly 140 million individuals in nearly fifty nations (Mochizuki, 2019). Abnormal blood arsenic values >0.01 μg ml^−1^ signify severe exposure however, they are only identified shortly after exposure (ATSDR, 2007). Arsenic metabolites are found in both organic as well as inorganic states, and they can be trivalent or pentavalent in oxidized form. As a result, there are several molecular species with various biological implications, further complicating diagnosis (Nicolis et al., 2009). Arsenate and arsenite, two dangerous inorganic pentavalent (As V) and trivalent (As III) arsenic species, have been linked to life-threatening complications, most notably CNS impairment (Jan et al., 2015). Cognitive deficits, developmental neurotoxic effects, and neurological conditions like Alzheimer’s and Parkinson’s disorder are all examples of this. Arsenate is thought to be physically similar to phosphate and capable of replacing it in biological functions and structures (Finnegan and Chen, 2012). It is absorbed into the regular phosphate pool in the body and eliminated at the same rate as phosphate i.e., excretion half-life of 12 days (Jan et al., 2015). Owing to its affinity for tissue proteins, levels in the blood are high for a brief period after exposure, following which it quickly disappears into different tissues (Abernathy and Morgan, 2001). Inorganic arsenic has a half-life of 4–6 h in blood, while methylated metabolites have a half-life of 20–30 h (Gebel, 1997). Long-term exposure to inorganic arsenic exacerbated Alzheimer’s like pathologies in the 3xTgAD mouse brain, including decreased Adenosine triphosphate (ATP) content and complex-I levels, and also increased ROS production in the hippocampus (Niño et al., 2018). Furthermore, greater immunopositive responses to amyloid isoforms and phosphorylated tau were seen in the frontal cortex and hippocampus (Cheng et al., 2021). Although not much is documented regarding the impact of different arsenic species on astrocytes *in-vivo*, primary culture experiments suggest that arsenic species are overtaken by astrocytes via an unknown pathway (Wang et al., 2012). In *Saccharomyces cerevisiae* and *Xenopus laevis* oocytes, GLUT1 facilitates arsenite absorption (Maciaszczyk-Dziubinska et al., 2012), but suppresses uptake of glucose and GLUT1 transport in human lymphocytes (Pánico et al., 2019). Arsenate and arsenite both have been found to promote Glutathione (GSH) export and induce glycolysis in astrocytes via an MRP1-mediated mechanism (Meyer et al., 2013; Tadepalle et al., 2014; Dringen et al., 2015). Because arsenic species stimulate astrocytic metabolism, this finding demonstrates that astrocytic metabolic disruption is a component of the mechanism *via* which arsenic species cause CNS impairments (De Keyser et al., 2008).

#### 2.3.3 Lead

Lead is a neurotoxin that passes the blood-brain membrane quickly, causing neuroinflammation, oxidative stress, endoplasmic reticulum stress, and apoptosis (Sanders et al., 2009). Despite regulatory attempts in the United States to reduce lead exposure, it is still used in industrial purposes such as vehicle lead-acid storage batteries (Dignam et al., 2019). Although the causes of lead exposure differ geographically, increased lead levels are often connected to electrical waste recycling, lead mining, and smelting, with inhalation and ingestion being the most common ways of exposure (Meyer et al., 2008). The existing limit for blood lead concentrations that are considered harmful (10 μg/dl) is substantially too high (Rossi, 2008). Based on the research findings, there is no safe level of lead in the blood, nonetheless, blood lead concentrations < 10 g/dl have been shown to have negative impacts on behavioural and cognitive functioning (Lanphear et al., 2000). Although lead has a short half-life in the blood, the time it takes for its detection in the blood can be much less than the time it takes for its harmful effects to manifest in the brain (Lidsky and Schneider, 2003). Reduced blood lead concentrations are not usually linked with possible lethal encephalopathy, they are neurotoxic in children and possess long-term consequences on neurobehavioural performance (Sanders et al., 2009). Lead poisoning from these lower levels of exposure is significantly more prevalent since there are no diagnostically clear visual indications (Wani et al., 2015). Because of the lack of obvious visible signs, such toxicity is commonly referred to as “asymptomatic,” but it is regrettably not “asymptomatic” when it comes to its consequences on cognitive ability (Lidsky and Schneider, 2003). Its exposure in children is extremely dangerous since lead dust is commonly ingested as a result of a children’s routine hand-to-mouth motion (Levin et al., 2008). Long-term research indicates that lead exposure throughout childhood or adolescence is associated with a higher risk of memory impairment (Finkelstein et al., 1998; Adlard et al., 2006). Lead exposure, whether prenatally or postnatally, promotes cognitive decline and loss in older animals (Kilian and Kitazawa, 2018). In human epidemiological research, it has been linked to a neurological disorder, and multiple investigations have found that its chronic or acute exposure leads to the characteristic indications of Alzheimer’s disease, including accumulation and inflammation (Monnet-Tschudi et al., 2006). Inflammatory processes that result in neuronal apoptosis can occur as a result of lead exposure (Adedayo et al., 2017). New studies suggest that lead exposure activates microglia (Kumawat et al., 2014) and causes excessive production of pro-inflammatory proteins like inducible nitric oxide synthase (iNOS), interleukin-1 beta (IL-1β), and tumor necrosis factor-alpha (TNF-α), which are largely attributed to neurodegeneration in Alzheimer’s disease (Zhang et al., 2017). In pregnant Wistar female rats, a 14-day (15 mg/kg) treatment of lead acetate (PbA) produced pro-inflammatory cytokines such as IL-1 and TNF- in the hippocampus and IL-6 in the forebrain of immature rat brain (Balali-Mood et al., 2021). Fortunately, via incorporating a range of modern treatments, lead toxicity can be remediated and lead concentrations in the body can be decreased (Patrick, 2006). Chelation therapy, nano-encapsulation, and N-acetylcysteine (NAC) are some of the most well-known treatments (Rathnakumr et al., 2019). Also, various antioxidants aid in the elimination of lead from the body (Hsu and Guo, 2002). Even though there are numerous therapeutic options accessible today, it is always preferable to avoid direct exposure to such substances and thereby deter possible effects (Wani et al., 2015).

#### 2.3.4 Manganese

Manganese is an essential minor element that is necessary for optimal growth, development, and cellular equilibrium. The normal concentration of manganese in body fluids ranges from 7 to 12 μgl^−1^ in blood, 0.6 to 4.3 μgl^−1^ in serum (Flora, 2014), and 1 to 8 μgl^−1^ in urine. The half-life of manganese in the blood is 10–42 days, whereas it is less than 30 h in urine (Flora, 2014). Manganese plays a vital role in osteogenesis, lipid, and carbohydrate metabolism, glycaemic management, and calcium absorption. It is an essential co-factor for various enzymes related to microglial cellular functions, as well as enzymes associated with neurotransmitter production and metabolic activity, in both humans as well as animals (Bowman et al., 2011). Manganese toxicity can occur as a result of occupational/environmental exposure, resulting in a neurodegenerative disorder called “Manganism” (Dobson et al., 2004). Manganese toxicity has shown an association with neurotoxicity and Parkinson’s-like disease characterized by motor, cognitive, and emotional impairment, which is thought to be caused by mitochondrial malfunction resulting in bioenergetic impairments (Harischandra et al., 2019). Moreover, the striatum of the basal ganglia (Tuschl et al., 2013) and globus pallidus are the major brain areas affected by elevated levels of manganese (Sidoryk-Wegrzynowicz and Aschner, 2013). Its exposure induces cell enlargement, a physical alteration seen in Alzheimer’s and hepatocerebral disorders (Balzano and El Hiba, 2019). It reacts with the astrocyte-produced enzyme such as pyruvate carboxylase (Sidoryk-Wegrzynowicz and Aschner, 2013), which performs a vital function in the tricarboxylic acid (TCA) cycle (a metabolic pathway used by aerobic organisms to produce cellular energy and intermediates for biosynthetic pathways) anaplerosis by catalyzing the carboxylation of pyruvate to oxaloacetate (Jitrapakdee et al., 2008). Consequently, manganese poisoning impairs both basic glial activity and astrocytic metabolic pathways resulting in primary as well as indirect neuronal impairment (Tjalkens et al., 2017).

#### 2.3.5 Synergistic Metal Toxicity

Metal neurotoxicity is commonly investigated based on metal type (Chen et al., 2016). Since, we live in a heterogeneous metal environment, which makes their research extremely difficult. Several metals, most notably manganese, zinc, cadmium, and copper are carried by common transporters or regulated via overlapping transmission mechanisms, fluctuations in one metal can have a significant influence on the stability of other metals (Zoroddu et al., 2019). Lead neurotoxicity in combination with other metals like arsenic, cadmium, mercury, and manganese has been widely investigated. Prenatal exposure to metals like lead and arsenic increases the risk of cognitive impairment compared to an individual metal (Karri et al., 2016). Lead levels beyond a certain threshold may have an impact on mental and psychomotor development in children exposed to increased cadmium levels during pregnancy (Sanders et al., 2015). Whereas exposure to increased concentrations of metals such as lead and manganese during pregnancy has revealed greater abnormalities in cognitive functioning in children at 2 years of age than exposed to an individual metal (Henn et al., 2014; Sanders et al., 2015). After being exposed to lead, children with elevated blood manganese concentrations have shown inferior intelligence quotient (IQ) scores (Roels et al., 2012). Given that the cumulative impact of methylmercury (MeHg) and lead exposure on intellectual impairments has been less than additive, lead with cadmium, manganese, or mercury exposure tends to work antagonistically, as opposed to the synergetic impact of lead and arsenic (Kumar et al., 2020). Studies suggest that the interaction of multiple metals, such as lead, mercury, aluminium, and arsenic, as well as the existence of specific genetic predispositions or epigenetic impacts, might cause autism spectrum disorder (ASD) symptoms (Bjørklund et al., 2018). Furthermore, recent studies found that Al and Hg had synergistic neurotoxic effects on primary human neuronal-glial (HNG) cells, resulting in a large upsurge in pro-inflammatory signalling mechanisms via notable induction of NF-kB (p50/p65) in response to Al and Hg individually or in combination (Alexandrov et al., 2018; Bjørklund et al., 2018). In numerous prevalent neurological conditions, fibrillar amyloid-like deposits and tau and α-synuclein co-deposits have been identified (Elimova et al., 2004; Carter, 2006). The most significant hazardous aggregation species, as per the new findings, are tiny oligomers (Afreen and Ferreira, 2022). Nübling et al. (2012) elucidated molecular crosstalks between distinct aggregation processes implicated in neurodegeneration and presented a novel viewpoint on interconnections among tau phosphorylation, metal ions, and the production of extremely hazardous oligomer species. The authors adopted confocal single-particle fluorescence to evaluate the effect of tau phosphorylation and metal ions on tau oligomer formation and co-aggregation with α-synuclein at the oligomer stage. Even at nano-molar protein levels, they revealed that Al^3+^ and tau phosphorylation via GSK-3 have synergistic impacts on the development of a unique SDS-resistant tau oligomer species. Tau phosphorylation, and also Al^3+^ and Fe^3+,^ increased both the production of blended oligomers and the incorporation of α-synuclein into pre-formed tau oligomers. Bimetal administration has been shown to augment the retention and re-alignment of single metals in rats. Selenium and mercury exposure enhanced both metal accumulation and caused mercury re-allocation in blood as well as organs. Betharia and Maher, (2012) reported that lower concentrations of metals such as lead, manganese, or a combination of the two showed gender-specific neurobehavioural implications. On postnatal days (24), manganese exposed males exhibited hypo-activity and showed higher anxiety as compared to controls, and similar tendencies were observed when the assay was repeated on postnatal days (59). Females and pups subjected to a metal combination, on the other hand, exhibited insignificant changes. Lead and manganese pharmacokinetic interactions were revealed by variations in blood, milk, and brain metal concentrations between samples taken from single metal and metal mixture exposed pups. Similarly, metal composites such as arsenic, manganese, and lead drastically reduced rat motor indices as compared to single-metal administration (Sanders et al., 2015). Since people are exposed to various metals at once in everyday life, and because neurotoxicity is frequently associated with overexposure of metals, further investigation into the health implications of metal combinations is recommended to comprehend their both synergetic as well as antagonistic impacts.

### 2.4 Pesticides

As per the epidemiological studies, most pesticides elevate the incidence of neurodegeneration (Sanchez-Santed et al., 2016). The most well-defined pathway for this link is mitochondrial toxicity, which leads to an upsurge in ROS (Re et al., 2008). In Parkinson’s disease, insoluble, misfolded proteins aggregate to form Lewy bodies, which are abnormal protein aggregations that develop inside the nerve cells severely affected by Parkinson’s disease (Breydo et al., 2012). While neurofibrillary tangles (NFTs) are hyperphosphorylated tau protein aggregates that are widely recognized as a basic biomarker of Alzheimer’s disease (Bengoa-Vergniory et al., 2021). Pesticide exposure can be minimized in school premises, workplaces, and residences by changing dietary habits and using sustainable pest management strategies (McBurney, 2017).

#### 2.4.1 Organophosphates

Pesticides containing organophosphates (OP) such as malathion, parathion, fenthion, dichlorvos, chlorpyrifos, diazinon, etc. are widely used worldwide (Satoh, 2006). Occupational/environmental exposures are one of the most common sources of contamination in people (Kapka-Skrzypczak et al., 2011). The primary issues of their exposure are the long-term consequences of both the high as well as low-level exposures over time, which have been linked to a higher incidence of chronic CNS disorders (Jamal et al., 2002) for instance exposure to chlorpyrifos (CPF) elevates the incidence of Alzheimer’s disease (Voorhees et al., 2019). Lee et al. (2016) demonstrated that regular administration of 3 and 10 mg/kg/d CPF induced 75–90% cortical AChE suppression, 40–80% hippocampus AChE suppression, and 90–100% whole blood cholinesterase (ChE) reduction, respectively, at the end of a 21 days of exposure in adult male rats (Long Evans). These treatments resulted in transcriptome alterations in genes producing hippocampal neuropeptides such as brain-derived neurotrophic factor (BDNF), cortistatin (CORT), and neuropeptide Y (NPY). In population subgroups like the elderly or genetically fragile groups, both lower and higher rates of exposure may have a notably significant effect (Sanchez-Santed et al., 2016). Organophosphate pesticides have a wide range of molecular targets, including hormones, neurotransmitters, neurotrophic variables, enzymes involved in the breakdown of β-amyloid protein, and proinflammatory alterations, in addition to their main activity of inhibiting the acetylcholinesterase enzyme (Sanchez-Santed et al., 2016).

#### 2.4.2 Organochlorines

Organochlorines belong to the category of chlorinated hydrocarbons with a multitude of uses and toxicity rates (Jayaraj et al., 2016). Organochlorine is used in pesticides, insecticides, herbicides, and a number of industrial applications such as plasticizers and coolants (Williams, 2011). Dichlorodiphenyltrichloroethane (DDT), rotenone, chlordane, dieldrin, aldrin, heptachlor, endrin, lindane, bornanes, and camphenes are some of the most well-known organochlorine insecticides. They vary in molecular structure, toxicity mechanism in target and non-target species, and a variety of other properties (Costa, 2015). Their activities and lethality do not always correspond with structure. In contrast, some structurally identical substances may have considerably different properties and toxic effects. For example, lindane (the γ-isomer of hexachlorocyclohexane) possesses insecticidal activities and stimulates the CNS, while others exhibit no such properties but still depress the CNS. They alter the role of sodium and calcium channels and transporters in the CNS and also interrupt γ-aminobutyric acid (GABA) neuro-transmission *via* obstructing certain GABA receptors, which adds to their neurotoxicity (Caudle, 2015). DDT and its associated substances remain in the environment and in animal tissues for a long period of time (How, 2009). Its exposure occurs *via* eating, inhaling, or touching products contaminated with DDT (Machino, 2016). DDT may transform into various metabolites, including dichlorodiphenyldichloroethene (DDE), and both remain in body and environment (Beard and Australian Rural Health Research Collaboration, 2006). Human symptoms such as vomiting, tremors/shakiness, seizures, etc. may result with high dosage exposure (How, 2009). Richardson et al. (2014) revealed that the increased serum DDE levels are linked with an elevated incidence of Alzhiemer’s disease and APOE4 ε4 allele carriers may be more prone to the effects of DDE. Both DDT and DDE enhance the level of amyloid precursor protein, lending mechanistic support to the link between DDE exposure and Alzhiemer’s disease. Rotenone is another commonly used insecticide (Lawana and Cannon, 2020) that has been associated with Parkinsonian symptoms like Lewy body development and nigrostriatal neurodegeneration (Uversky, 2004). It causes nigrostriatal degeneration in clinical models of Parkinson’s disease by elevating intracellular calcium and activating phosphorylation and aggregation of α-synuclein (Yuan et al., 2015). Paraquat is a highly hazardous quarternary nitrogen herbicide (Tan et al., 2013). Its long-term exposure has been implicated as a risk factor for Parkinson’s disease (Tamano et al., 2019). Paraquat causes modifications in α-synuclein and enhances α-synuclein aggregation (Rokad et al., 2017). Paraquat caused Parkinsonism in mice via promoting oxidative stress, degenerative cellular apoptosis in the substantia nigra pars compacta (SNpc), striatum, and cerebellum, as well as dopamine deficiency in the SNpc and striatum, which led to motor and cognitive impairments (Onyeka et al., 2022). Prenatal and postnatal organochlorine exposure has been linked to neurodevelopmental disorders such as poor psychomotor development, memory loss, anxiety, autism, etc. (Saravi and Dehpour, 2016). Adult female CD1 mice treated for 20 days with sesame oil (vehicle) or methoxychlor (MXC) at doses of 16, 32, or 64 mg/kg/day showed dose-related declines in striatal dopamine levels, which were preceded by decreased levels of the dopamine transporter, vesicular monoamine transporter-2, and elevated protein carbonyl levels in non-synaptic mitochondria, indicating a close association between mxc and a higher likelihood of Parkinson’s disease (Schuh et al., 2009). Furthermore, chlorination improves a hydrocarbon’s solubility and blood-brain membrane penetration, making these molecules strong CNS infiltrators (McCann and Maguire-Zeiss, 2021).

### 2.5 Polychlorinated Biphenyls (PCBs)

Polychlorinated biphenyls are a broad category of organochlorines with different modes of action based on the level and placement of the chlorine atoms on the biphenyl rings (Adetutu et al., 2020). These were manufactured globally from around 1930 until they were limited by the Stockholm Convention on Persistent Organic Pollutants (POPs) in 2001, despite prior prohibitions issued by various institutions, such as the US-EPA and the Eu-Commission (El-Shahawi et al., 2010). Despite these restrictions, they are still present in the environment owing to a variety of biochemical aspects, like lipid solubility and lengthy half-lives. PCBs mostly accumulate in fatty organs, namely the brain, where their presence has been related to a variety of disorders and symptoms, notably Parkinson’s disorder and cognitive impairment (Gagnon-Chauvin et al., 2020). Lyng et al. (2007) used an organotypic co-culture system of developing rat striatum and ventral mesencephalon (VM) to study the impact of PCBs on the developing basal ganglia Dopamine (DA) system VM. The co-cultures were exposed to an environmentally relevant blend of PCBs for 1, 3, 7, and 14 days, causing changes in DA neurochemistry, DAergic protein downregulation, and elevated oxidative stress, which led to the extraneuronal injury and the eventual deterioration of both VM and striatal GABA neurons, prior to the death of VM DA neurons. The authors suggested that the co-culture technique was an effective model for studying the sequence of PCB-induced neurotoxic events in developing basal ganglia and that it could have significant implications for conditions including Parkinson’s disease, as well as cognitive impairments associated with PCB exposure and toxicologically identical environmental toxins. Wang et al. (2022) studied the toxicity of a lab-processed blend of PCBs intended to mimic an indoor school environment on female Sprague-Dawley rats via nose-only exposure techniques, incorporating transcriptomics, metabolomics, and neuro-behavioural outcomes. Its exposure has been proven to impair memory, cause anxiety-like behaviour, substantially lower white blood cell counts, slightly affect plasma metabolomics, and impact transcription brain activity, with 274 genes upregulated and 58 genes downregulated. Although they have been related to oxidative stress and cellular dysfunction in several *in-vivo* as well as *in-vitro* models their effect on astrocytes has received less attention (McCann et al., 2021).

### 2.6 Solvents

Solvents are volatile substances, and most environmental exposures are caused by inhaling solvent vapour (Dick, 2006). Organic solvents are used extensively in industries all over the world. Paints, medicines, lubricants, printing inks, insecticides, cosmetics, and home cleansers all require organic solvents, which are omnipresent in today’s society (Ciriminna et al., 2014). Isopropanol, toluene, xylene, solvent mixes like white spirits, and chlorinated solvents like methylene chloride, trichloroethylene, and perchloroethylene are all extensively employed solvents (Gupta, 2020). The coatings sector is the greatest major consumer, as solvents are critical to the quality and longevity of paints, adhesives, varnishes, etc (Dick, 2006). Organic solvent usage is falling in several industries, such as laundry detergent, owing to advances in machinery and processes. Solvents are progressively being recovered and recycled, mainly as a result of environmental restrictions on volatile organic compound emissions (Gani et al., 2006). 1-Bromopropane, a solvent capable of replacing ozone-depleting chemicals like 1,1,1trichloroethane (methyl chloroform), has recently been found to possess neurotoxicity in humans (Saygun et al., 2012). Trichloroethylene (TCE), a chlorinated halocarbon organic solvent, is a mitochondrial toxin associated with dopaminergic neurodegeneration (Castro et al., 2020). Solvents such as n-hexane, methyl n-butyl ketone, 2,5-hexanedione, acetone, methyl ethyl ketone, methyl isobutyl ketone, carbon disulphide, styrene, and 1,1,1-trichloroethane have been associated with peripheral neurotoxins (Xiao and Levin, 2000). De Miranda et al. (2021) studied whether long-term and systemic trichloroethylene exposure at a 200 mg/kg dose activated wild-type LRRK2 and resulted in nigrostriatal dopaminergic impairment dose in aged rats. The authors proposed a gene-environment link between ambient mitochondrial toxins and the protein kinase LRRK2 because it increased LRRK2 kinase activity in the brain, causing a severe dopaminergic lesion in the nigrostriatal area, increased oxidative stress, and induced endolysosomal impairment, and α-synuclein deposition. Chronic toxic encephalopathy, psycho-organic syndrome, or solvent neurotoxicity are all terms used to describe a syndrome of changes in personality, cognitive impairment, and neuropathies caused by chronic, high-level solvent exposure (Spee et al., 2012).

## 3 Common Mechanism of Environmental Neurotoxicity

Nearly all neurological disorders have significant pathways in common. Blood-brain barrier disruption, protein aggregation, oxidative stress, and mitochondrial impairment are some key pathogenic processes that occur together (Jellinger, 2010). Neurotoxicants can either trigger or accelerate such processes, resulting in neurodegeneration (Cannon and Greenamyre, 2011).

### 3.1 Blood-Brain Barrier Disruption

The vascular endothelium in the brain is a vital component of the blood-brain-barrier (BBB) due to its highly compact structure, which helps to maintain a functional and molecular barrier between the brain and the rest of the body, as well as to shield neurons from infections and toxins (Noe et al., 2020). Simultaneously, the BBB coordinates molecular transportation in and out of the CNS. BBB disruption and failure in cerebrovascular disorders cause component leakage into the CNS, leading to neurodegenerative impairments (Sweeney et al., 2019). BBB dysfunction has been found in neurodegenerative conditions such as multiple sclerosis, Parkinson’s, and Alzheimer’s disease (Zhao et al., 2015). Neuronal cells are highly susceptible to pro-inflammatory cytokines such as IL-1, IL-6, TNF-α, lipid mediators, free radicals, vasogenic agents such as glutamate, serotonin, and histamine, and other endogenous stimuli such as extracellular K^+^ and intracellular Ca^2+^ (Muralidharan and Kofke, 2016). Most of them are produced under pathophysiological circumstances, and alterations in their levels in the BBB play an important role in the genesis and progression of CNS impairment (Gonzalez-Candia et al., 2021). Li et al. (2020) studied indoor nanoscale particulate matter (INPM)-induced BBB disruption and possible cellular responses using a 3D human organotypic model. Human astrocytes and human umbilical vein endothelial cells were co-cultured in 3D within a microfluidic system to replicate the micro-complex physiological responses of the BBB to INPM exposure. INPM exacerbated inflammation caused by ROS and stimulated abnormal expression of the nuclear transcription factor Nrf2 following ROS accumulation. This activity disrupted the synthesis of γ-glutamate synthase (γ-GCS) and heme oxygenase (HO-1), exacerbating the antioxidant system’s imbalance and thereby influencing BBB bio-function by Keap1-Nrf2-ARE pathways. According to Liu et al. (2020) triphenyl phosphate (TPP) and its metabolite diphenyl phosphate (DPP) produced metabolomic and transcriptome changes in the brain. TPP (0, 50, or 150 mg/kg/day) was administered orally to weaned male mice (C57/BL6) for 30 days. The thalamus and hippocampus of mice exposed to high doses showed signs of inflammation, as well as changes in glutamic acid, N-acetyl CoA metabolites, and organic acid levels. TPP exposure seemed to interfere with amino acid and lipid metabolism, as well as brain transcription and cell death processes (FOXO and MAPK signalling pathways). Anti-inflammatory cytokines such as TNF-α and interleukin-6 (IL-6) were upregulated, but antioxidant genes such as nuclear factor-E2-related genes were downregulated.

### 3.2 Protein Aggregation

**FIGURE 2:**
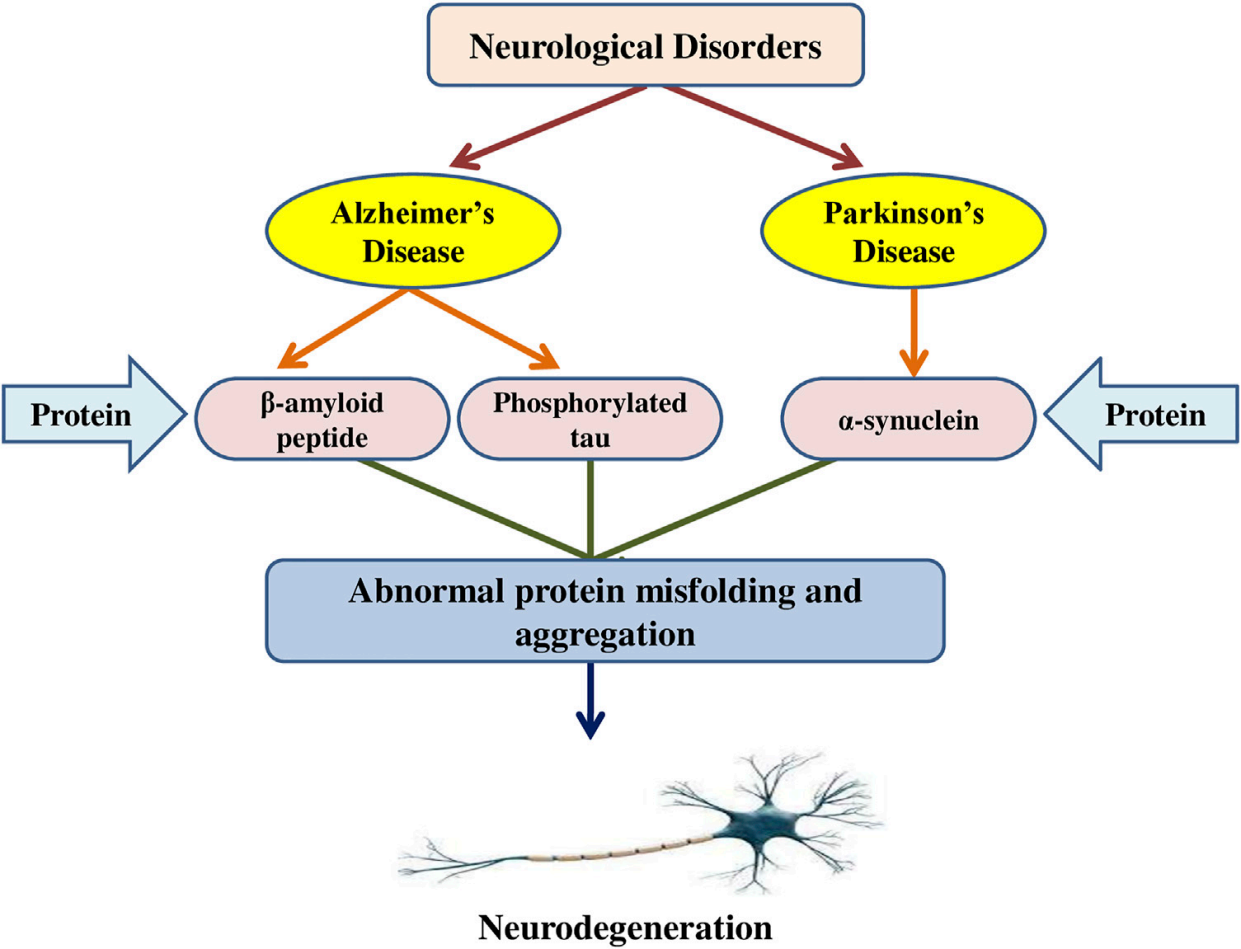
The mechanism of protein aggregation in neurodegenerative disorders.

Misfolded protein aggregates are a typical histopathological feature in various neurological disorders (Lázaro et al., 2020). Aggregates can result in the formation of disease-specific protein alterations that make them prone to aggregation or elevate their cellular level (Guo and Lee, 2014). Furthermore, environmental parameters and the impacts of ageing perform major functions. The potential to maintain cellular proteostasis, in particular, decreases considerably with age. There are two types of aggregates: 1) tiny, soluble oligomeric aggregates that can evolve into 2) larger insoluble protein aggregates termed inclusion bodies (IBs), which have been found to adopt amyloidogenic and amorphous forms (Bäuerlein et al., 2020). The existence of tiny soluble oligomeric aggregates is linked to toxicity in several conditions, such as Alzheimer’s, Parkinson’s, and other neurodegenerative disorders, and IB production is frequently seen as a protective strategy (Vendredy et al., 2020). Furthermore, in addition to oligomers, IBs have been found to have significant cytotoxic activities in these disorders for α-synuclein, Tau, TAR DNA-binding protein 43 **(**TDP-43), and β-amyloid. IBs carrying α-synuclein have been identified in Parkinson’s disease, Lewy-body disease, etc. (Stefanis, 2012). Hyperphosphorylated tau protein precipitates cytosolically as neurofibrillary tangles are observed in IBs of Alzheimer’s disease (Bäuerlein et al., 2020). TDP-43 accumulates in IBs observed in amyotrophic lateral sclerosis (ALS) and frontotemporal dementia, but also Lewy-body disease, Alzheimer’s, Parkinson’s, and other diseases. β-amyloid is the primary component of the extracellular neuritic plaques observed in Alzheimer’s diseases (Jansen et al., 2014). Although the IBs of diverse neurological conditions are produced via distinct proteins, they are usually cross β-sheet amyloid fibrils. Inside a neuron, axonal damage and protein aggregation promote an equivalent reactive pathway (Serrano-Pozo et al., 2011). Aggregates like β-amyloid might indicate damage or produce physical deformity or damage to structural proteins like tau and neurofilament (NF) triplet proteins, which subsequently initiate a sequence of cytoskeletal alterations leading to dystrophic neurite production and the eventual onset of apoptosis (Vickers et al., 2000). Protein aggregates may quicken the aggregation process via binding with pathogenic proteins such as chaperones. In glia, αB-crystallin (a chaperone protein) binds to the unfolded proteins and suppresses protein aggregation (Segura-Aguilar and Kostrzewa, 2006). Furthermore, cytoplasmic protein aggregates are currently considered to have a neuro-protective effect by sequestration and neutralization of harmful peptides and proteins (Mangione et al., 2016). The interaction between α-synuclein oligomers and lipid membranes (Musteikytė et al., 2021) or parts of the ubiquitin-proteasome system is hypothesized to cause toxicity (Cook and Petrucelli, 2009). Moreover, the lipid-soluble component of α-synuclein is elevated in Alzheimer’s disease (Larson et al., 2012). Metallothioneins are cytoplasmic proteins that are thought to be neuroprotective since they bind heavy metals, have antioxidant and antiapoptotic properties and reduce cellular inflammation (Zalewska et al., 2014). Advanced glycation end products (AGEs) are age-related protein aggregates seen in amyloid plaques, neurofibrillary tangles, and α-synuclein that develop when reactive dicarbonyls like methylglyoxal combine (Krautwald and Münch, 2010). Srivastava et al. (2020) evaluated the toxicity of α-synuclein conformers on neuronal SH-SY5Y cells in the presence of rotenone via MTT test, Annexin-V apoptosis assay, ROS detection assay, and the mitochondrial membrane potential evaluation. The findings revealed that rotenone increased the production of structurally unique oligomers and fibrils that function as templates, as well as enhanced the development of conformers likely to spread to nearby neuronal cells. Furthermore, the involvement of the NAC region and the helical conformations formation resulted in structural alterations in oligomers and fibrils, which affects their cytotoxic activity, where β-sheet rich oligomers and fibrils modify the membrane potential of neuronal cells and cause early cell death. The mechanism of protein aggregation in neurodegenerative disorders is presented in Figure 2.

### 3.3 Mitochondrial Impairment and Oxidative Stress

**FIGURE 3:**
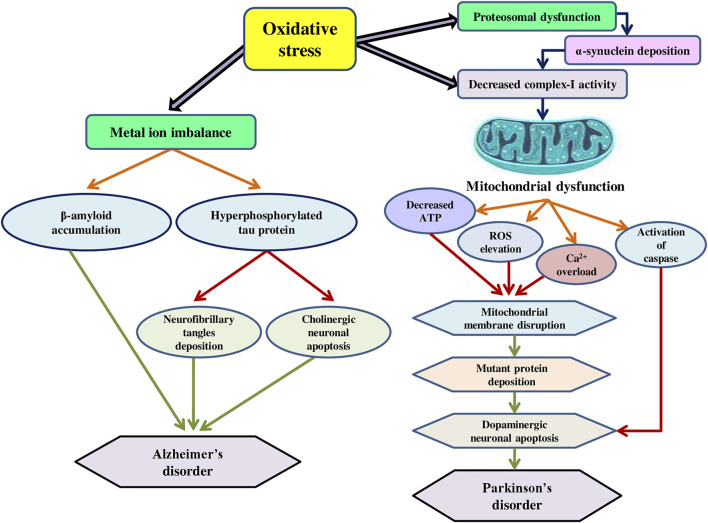
The mechanism of mitochondrial dysfunction and oxidative stress in Alzheimer’s and Parkinson’s disorders.

The development of neurodegenerative conditions, such as Alzheimer’s, Parkinson’s, and other related disorders have been linked to both mitochondrial dysfunction and oxidative stress (Elfawy and Das, 2019). Severe oxidative stress to lipids, proteins, and DNA characterizes these disorders, which can trigger apoptosis through various pathways, including the deactivation of critical functions or the activation of fatal processes (Finkel and Holbrook, 2000). Oxidative stress is produced by a loss of balance in the pro-oxidant/antioxidant equilibrium, resulting in the formation of deleterious ROS (Guerin et al., 2001). ROS are specific to the brain and cause oxidative stress and are highly active in the brain and neuronal tissue as excitative amino acids and neurotransmitters, whose metabolism is a producer of ROS (Gilgun-Sherki et al., 2002). They target post-mitotic cells like glial cells and neurons, which are more vulnerable to free radicals, causing neurological injury (Uttara et al., 2009). Furthermore, ROS build-up and oxidative stress might be caused by impaired ROS removal capabilities. Redox imbalance is caused by a decrease in the quantity of GSH, an essential intracellular antioxidant, in various conditions, such as Parkinson’s disorder, cystic fibrosis, and others (Ballatori et al., 2009). Mitochondria in association with the endoplasmic reticulum are involved in calcium regulation, inorganic ROS production, and lipid signalling and metabolism (Rana, 2019). Furthermore, they are a major source of amyloid and tau accumulation in Alzheimer’s disorder in brain tissue (Ferreiro et al., 2012). Oxidative stress affects dopaminergic neurons, cholinergic receptors, and a variety of other structures implicated in neurodegeneration, triggering a chain of events that includes mitochondrial malfunction and neuroinflammation (Rekatsina et al., 2020). As a result, the degeneration of neurons is aided by the damage of nuclear and mitochondrial DNA (mtDNA) (Mandavilli et al., 2002). Since neurons have high energy requirements, mitochondrial oxygen consumption accounts for over 90% of mammalian oxygen consumption. Moreover, complex-I inhibition reduces mitochondrial ATP synthesis and promotes the formation of ROS, which in turn damage mitochondrial DNA, respiratory chain components, and other mitochondrial factors, resulting in a deadly cycle of mitochondrial malfunction and oxidative stress (Exner et al., 2012). Adult male Sprague Dawley rats were administered mitochondria-targeted antioxidant (Mito-Q (250 μM)) in drinking water for 4 weeks, followed by simulated vehicle exhaust exposure (SVEE) for 2 weeks (5 h/day), and then behavioural and biochemical tests were performed. The findings revealed that VEE caused behavioural and cognitive abnormalities, elevated oxidative stress, a decreased antioxidant response, and mitochondrial impairment as a result of electron transport chain (ETC) disruption, decreased oxygen consumption, low ATP synthesis, and a modification in mitochondrial biochemical dynamics as measured by protein expression profiles of mitochondrial fission, dynamin-related protein-1, and fusion markers, mitofusin-1/2 in the hippocampus (HIP), amygdala (AMY) and the prefrontal cortex (PFC). Mito-Q administration reduced behavioural abnormalities, oxidative stress, and averted mitochondrial damage caused by SVEE (Salvi et al., 2020). Kaur et al. (2007) studied the effect of dichlorvos (organophosphate) on mitochondrial calcium uptake, oxidative stress generation, and neuronal death using an *in-vivo* rat model. Dichlorvos was given subcutaneously at a dose of 6 mg/kg/day for 12 weeks, and the results showed a substantial increase in mitochondrial Ca^2+^ absorption, reduced cytochrome oxidase (complex-IV) electron transfer activities, and modified mitochondrial complex-I, and complex-II activity. Furthermore, reduced GSH levels and manganese superoxide dismutase (Mn-SOD) activity in mitochondria isolated from dichlorvos-treated rat brain induced an increase in malondialdehyde, protein carbonyl, and 8-hydoxydeoxyguanosine synthesis, as well as protein and mtDNA oxidation as a result of elevated oxidative stress. Chronic low-level dichlorvos exposure resulted in oligonucleosomal DNA fragmentation, a characteristic sign of apoptosis. The mechanism of mitochondrial dysfunction and oxidative stress in Alzheimer’s and Parkinson’s disorders is presented in Figure 3. Mitochondria are a potential therapeutic target in neurological disorders due to their neuroprotective effect when functioning effectively (Camara et al., 2017). Although it is a new area of study, there are presently no treatments in clinical trials that specifically target mitochondrial quality control. Previous therapies aiming at correcting mitochondrial dysfunction failed to produce the desired outcomes, in part due to the delays of the intervening window (Almannai et al., 2020).

## 4 Conclusion and Future Perspectives

Owing to the escalating dementia incidence and growing environmental pollution across several geographic locations throughout the world, the developing link connecting to the exposure of many occupational/environmental hazardous chemicals and neurological disorders is of great public health relevance. Several epidemiological investigations illustrate inconsistent findings in estimating the risk level for both Alzheimer’s and Parkinson’s disorders. Due to the number of drawbacks, including the complexity in precisely diagnosing Alzheimer’s or Parkinson’s disease cases leading to lack of biochemical markers, the inability to reliably evaluate prolonged exposures, and the omission of vital influencing factors, for instance, co-exposure to toxicants, genetic variants, and lifestyle, etc. Nonetheless, epidemiological research and observational results have pointed to the possible hazard of developing these neurodegenerative disorders as a result of exposure to environmental toxins including heavy metals and pesticides. Remarkably, both pollutants have comparable toxicity pathways, which converge in a generalized process aimed at the emergence of oxidative stress, which results in both neurological diseases characteristics. Furthermore, the majority of research has not factored in the speciation of these toxins, and the evaluation of exposure via both drinking water and food is not adequately documented. Specifically, when an individual has a neurological disorder that impairs cognitive ability, assessing recollected exposure is more challenging. Some epidemiological studies imply a link between neurological conditions and metals such as aluminium in water, however other research does not substantiate this link. Most of the studies lack knowledge on metal uptake via food and how toxin levels in food alter the link between toxins in water and neurodegenerative diseases. More analytical studies are required to identify whether these toxins from diverse sources exhibit a major causative link with Alzheimer’s, Parkinson’s, and other neurodegenerative disorders. Additionally, the relationship between early-life exposure to environmental variables and the onset of neurological illnesses is gaining traction, which might assist to elucidate the function of the environment in the progression of such disorders. On the other side, the absence of particular biomarkers for Alzheimer’s and Parkinson’s disorders restricts early detection and therapy. Furthermore, identifying biomarkers to assess previous exposure to environmental contaminants is critical for effective and timely care of such disorders. Therefore, as we gain a better understanding of the risks associated with environmental contaminant exposures, more detailed epidemiological investigations are required to raise the standard of life of the aged and to avoid the onset of neurological disorders across the world.

## Glossary

NFTs: Neurofibrillary Tangles
Pb: Lead
Al: Aluminium
Hg: Mercury
Mn: Manganese
Cd: Cadmium
As: Arsenic
CNS: Central Nervous System
IQ: Intelligence Quotient
iNOS: Inducible Nitric Oxide Synthase
IL-1β: Interleukin-1 beta
TNF-α: Tumor Necrosis Factor-alpha
NAC: N-acetylcysteine
GSH: Glutathione
TCA: Tricarboxylic Acid Cycle
OP: Organophosphates
CPF: Chlorpyrifos
AChE: Acetylcholinesterase Enzyme
ChE: Cholinesterase
TDP-43: TAR DNA-binding protein 43
VM: Ventral MesencephalonF
DA: Dopamine
SVEE: Simulated Vehicle Exhaust Exposure
ETC: Electron Transport Chain
ATP: Adenosine Triphosphate
Mn-SOD: Manganese Superoxide Dismutase
Mito-Q: Mitochondria-targeted antioxidant
HIP: *Hippocampus*
AMY: Amygdala
PFC: Arefrontal Cortex
INPM: Indoor Nanoscale Particulate Matter
TPP: Triphenyl Phosphate
γ-GCS: γ-Glutamate Synthase
HO-1: Heme Oxygenase
mtDNA: Mitochondrial DNA
SNpc: Substantia Nigra Pars Compacta
SPF: Specific-Pathogen-Free
PCBs: Polychlorinated biphenyls
POPs: Persistent Organic Pollutants
TCE: Trichloroethylene
ASD: Autism Spectrum Disorder
HNG: Human Neuronalglial
BBB: Blood Brain Barrier
ROS: Oxygen Reactive Species
AlCl3: Aluminium chloride
Al(lac)3: Aluminium lactate
IBs: inclusion bodies
NF: Neurofilament
ALS: Amyotrophic Lateral Sclerosis
AHEI: Alternate Healthy Eating Index
aMed: alternate Mediterranean Diet Score
AGEs: Advanced glycation end products
BDNF: Brain-derived neurotrophic factor
CORT: Cortistatin
NPY: Neuropeptide Y
MXC: Methoxychlor
WHO: World Health Organization
